# Improvement of Social Isolation and Loneliness and Excess Mortality Risk in People With Obesity

**DOI:** 10.1001/jamanetworkopen.2023.52824

**Published:** 2024-01-22

**Authors:** Jian Zhou, Rui Tang, Xuan Wang, Xiang Li, Yoriko Heianza, Lu Qi

**Affiliations:** 1 Department of Epidemiology, Tulane University School of Public Health and Tropical Medicine, New Orleans, Louisiana; 2 Department of Orthopedics, The Second Xiangya Hospital of Central South University, Changsha, China; 3 Department of Nutrition, Harvard T.H. Chan School of Public Health, Boston, Massachusetts

## Abstract

**Importance:**

Individuals with obesity experience markedly higher levels of social isolation and loneliness than those without obesity, but little is known about whether improvement of social isolation or loneliness might attenuate obesity-related excess risk of mortality.

**Objective:**

To investigate whether improvement of social isolation or loneliness is associated with lower obesity-related excess risk of mortality.

**Design, Setting, and Participants:**

This cohort study included individuals without cancer or cardiovascular disease (CVD) at baseline from the UK Biobank with follow-up beginning in March 2006 and ending in November 2021.

**Main Outcomes and Measures:**

All-cause, cancer-related, and CVD-related mortality were estimated.

**Results:**

A total of 398 972 participants were included in this study (mean [SD] age, 55.85 [8.08] years; 220 469 [55.26%] women; 13 734 [3.44%] Asian, 14 179 [3.55%] multiracial, and 363 685 [91.16%] White participants). Overall, 93 357 (23.40%) had obesity, and 305 615 (76.60%) did not. During a median (IQR) follow-up of 12.73 (12.01-13.43) years, a total of 22 872 incident deaths were recorded. Compared with participants with obesity with an index of 2 or greater for social isolation, the multivariable adjusted hazard ratios (HRs) for all-cause mortality were 0.85 (95% CI, 0.79-0.91) and 0.74 (95% CI, 0.69-0.80) for participants with obesity and a social isolation index of 1 and 0, respectively (*P* for trend < .001); compared with participants with obesity and an index of 2 for loneliness, the HRs and 0.97 (95% CI, 0.89-1.06) and 0.86 (95% CI, 0.79-0.94) for participants with obesity and a loneliness index of 1 and 0, respectively (*P* for trend < .001). As the index of social isolation and loneliness went from highest to lowest, the HR for all-cause mortality decreased by 36% and 9%, respectively, in people with obesity compared with people without obesity using the multivariable model. Social isolation was ranked higher than loneliness, depression, anxiety, and lifestyle-related risk factors including alcohol, physical activity, and healthy diet for estimating the risks of all-cause mortality, cancer-related mortality, and CVD-related mortality.

**Conclusions and Relevance:**

In this cohort study of UK Biobank participants, a lower index of social isolation or loneliness was associated with a decreased risk of all-cause mortality among people with obesity, and improvement of social isolation and loneliness attenuated obesity-related excess risk of all-cause mortality.

## Key Points

**Question:**

Could the excess risk of mortality related to obesity be attenuated through the improvement of social isolation and loneliness?

**Findings:**

In this cohort study of 398 972 UK Biobank participants, as the index of social isolation and loneliness went from highest to lowest, the risk of all-cause mortality decreased by 36% and 9%, respectively, in people with obesity compared with people without obesity. Social isolation ranked higher than loneliness, depression, anxiety, and lifestyle-related risk factors for estimating the risk of mortality.

**Meaning:**

These findings support the improvement of social isolation and loneliness in people with obesity to decrease obesity-related excess risk of mortality.

## Teaser

This cohort study investigates whether improving social isolation or loneliness is associated with lower obesity-related excess risk of mortality.

## Introduction

Obesity is a pervasive and escalating global concern. In high-income countries, approximately 30% of the general population is classified as having obesity.^1^ Obesity has been consistently related to excess risks of all-cause mortality and mortality due to cardiovascular disease (CVD) and cancer in various populations.^2^

Efforts to tackle the issues of social isolation and loneliness have been simmering for decades.^3^ The US Surgeon General has highlighted an urgent need to confront the public health crisis posed by loneliness.^4^ In our recent work,^5^ we observed a significant association between loneliness and risk of CVD in patients with diabetes. Mounting evidence shows that people with obesity encounter markedly higher levels of social isolation and loneliness than those without obesity.^6,7,8^ Social isolation and loneliness are crucial aspects of social determinants of health that represent distinct components of social contact.^9,10^ Social isolation refers to the amount of social interaction observed in behavior, while loneliness typically pertains to emotional experiences linked to social relationships’ quality.^11,12^ Previous studies on the general population have found that social isolation^13,14^ and loneliness^15,16^ were significantly associated with elevated risks of mortality. We hypothesized that improvement of isolation and loneliness would be associated with a reduction of obesity-related excess risk of mortality. To our knowledge, no prospective study has yet addressed such a hypothesis.

In this study, our aim was to examine the associations of improvement of social isolation and loneliness with the risk of mortality in individuals with obesity or without obesity. We also compared people with obesity with matched control participants without obesity to investigate whether the obesity-related excess risk of mortality could be attenuated or eliminated through improving social isolation and loneliness indexes. Additionally, we sought to compare the significance of social isolation and loneliness against lifestyle-related risk factors for mortality.

## Methods

This cohort study followed the Strengthening the Reporting of Observational Studies in Epidemiology (STROBE) reporting guideline. The UK Biobank study was approved by the National Health and Social Care Information Management Board and the North West Multicenter Research Ethics Committee and the institutional review board of Tulane University. All participants provided written informed consent at recruitment to the study.^17^

### Study Population

The UK Biobank is a large-scale prospective population-based cohort study with more than 500 000 participants aged 40 to 70 years between 2006 and 2010.^17^ The obesity and nonobesity groups were defined as body mass index (BMI; calculated as weight in kilograms divided by height in meters squared) of 30 or greater and less than 30, respectively.^18^ In this cohort study, we included 93 357 people with obesity and 305 615 people without obesity. A total of 84 920 people with obesity were then matched for age, sex, and assessment center with 1 control participant without obesity randomly, and 84 920 matched control participants were enrolled in this study (eFigure 1 in Supplement 1).

### Definition of Social Isolation and Loneliness Scales

The social isolation and loneliness indexes were constructed using self-reported questionnaires in UK Biobank^12,19^ (eTable 1 in Supplement 1). The social isolation and loneliness index were assessed via 3 and 2 questions, respectively. Given the relatively small number of participants in with social isolation index scores of 3, participants with an index of 3 were combined into index of 2 or greater group. We calculated the total indexes of social isolation and loneliness by summing the individual indexes of the 3 and 2 corresponding indicators, respectively, with a range of 0 to 2 or greater and 0 to 2. Detailed information of scoring methods for social isolation and loneliness are indicated in eTable 1 in Supplement 1.

### Outcomes

The primary results of this study included mortality from all causes, cancer, and CVD. The end date for follow-up was determined as the date of the baseline to death or the censoring date (November 27, 2021), whichever occurred first. The results were categorized according to the *International Statistical Classification of Diseases and Related Health Problems, Tenth Revision (ICD-10)* . For the current study, we analyzed mortality for all-cause, cancer (codes C00 to C97), and CVD (codes I00 to I99).^20,21^

### Other Variables

Age, sex, race and ethnicity, Townsend Deprivation Index, education years, smoking status, alcohol intake, and hemoglobin A_1c_ (HbA_1c_) level were obtained directly from UK Biobank. The healthy diet score (eTable 2 in Supplement 1) was created according to our previous studies.^22,23^ We classified participants into 2 groups based on their total moderate physical activity minutes per week, following global recommendations for physical activity and health.^24^ One minute of vigorous physical activity was considered equivalent to 2 minutes of moderate physical activity. The 2 groups were defined as follows: less than 150 or 150 or more minutes per week. Depression, anxiety, and eating disorders were defined as having a self-reported history or being diagnosed using *ICD-10* codes^25^ (eTable 3 in Supplement 1). We defined hypertension as having a self-reported history of hypertension, a systolic blood pressure equal to or greater than 140 mm Hg, a diastolic blood pressure equal to or greater than 90 mm Hg, taking antihypertensive medications, or being diagnosed using *ICD-10* codes. High cholesterol was defined as having a self-reported history of high cholesterol, taking cholesterol-lowering medications, or being diagnosed using *ICD-10* codes. Diabetes was defined as having a self-reported history of diabetes, using insulin, or being diagnosed using *ICD-10* codes (eTable 3 in Supplement 1). Metformin use and glucocorticoid use were defined as self-reported drug use^26^ (eTable 4 in Supplement 1). The details of these variables can be found on the UK Biobank website.^27^

### Statistical Analysis

Continuous variables were presented as mean and SD and categorical variables were presented as counts with percentages. We used Fisher exact tests or Wilcoxon rank-sum tests to examine participant characteristics according to whether participants had obesity. We used Cox regression models to observe the association of social isolation and loneliness with risk of all-cause mortality, cancer-related mortality, and CVD-related mortality in people with obesity. The validity of the proportional hazards assumption was assessed by utilizing Schoenfeld residuals and Kaplan-Meier methods, and all evaluations satisfied the predetermined criteria. The group with the highest index was set as the reference group. The basic model was adjusted for age (years) and sex (male or female). The multivariable model was further adjusted for race and ethnicity (Asian, Black, Chinese, multiracial, White, or other [not further defined in UK Biobank]), Townsend Deprivation Index (continuous), years of education(<15, 15-19 or ≥20 years), smoking status (never, previous, or current smoking), alcohol intake (<3 or ≥3 times/wk), healthy diet score (<3 or ≥3), physical activity (≥150-min/wk or <150-min/wk), depression (yes or no), anxiety (yes or no), eating disorders (yes or no), hypertension (yes or no), high cholesterol (yes or no), diabetes (yes or no), metformin use (yes or no), glucocorticoid use (yes or no) and HbA_1c_ level (continuous). Then we used the same Cox models when comparing people with obesity with matched control participants. We coded the missing data of categorical covariates and continuous variables using a missing indicator category and mean values respectively. The numbers and percentages of participants with missing covariates are shown in eTable 5 in Supplement 1. eFigure 2 in Supplement 1 is a directed acyclic graph explaining the associations between the exposures, the outcome, and the covariates.

We further categorized the social isolation status as social isolation (social isolation index ≥2) and non–social isolation (social isolation index <2).^19,28,29^ Similarly, the loneliness status was split into 2 groups: loneliness (loneliness index of 2) and nonloneliness (loneliness index <2).^19,28,29^ To calculate the contribution of social isolation, loneliness, and covariates to the explained relative risk derived from the multivariable model, we used the coxphERR function^30^ from Rstudio version 4.1.2 (R Project for Statistical Computing) to observe the relative importance of risk factors in people with obesity.

Two sensitivity analyses were conducted to explore the stability of the results. Participants who died during the first 2 years of follow-up were excluded. Then, all missing covariate data were inputted using chained equations. All results were expressed as the hazard ratio (HR) and 95% CI. SAS version 9.4 (SAS Institute) was used to perform the statistical analysis, and we considered a 2-sided *P* < .05 as indicating statistically significant differences.

## Results

### Baseline Characteristics of Participants

Of the 398 972 included participants (mean [SD] age, 55.85 [8.08] years; 220 469 [55.26%] women; 13 734 [3.44%] Asian, 14 179 [3.55%] multiracial, and 363 685 [91.16%] White participants), 93 357 participants (23.40%) had obesity, and 305 615 (76.60%) did not. Among 93 357 people with obesity, 45 673 (48.92%), 37 881 (40.58%), and 9803 (10.50%) participants were defined as having a social isolation index of 0, 1, and 2 or greater, respectively. The corresponding percentage was 59 342 (63.56%), 26 627 (28.52%), and 7388 (7.91%) for participants with obesity and loneliness index of 0, 1, and 2. The prevalence of social isolation and loneliness in people with obesity was significantly higher than that in people without obesity (*P* < .001) (Table 1).

**Table 1.** Baseline Features of Participants

Characteristic	Participants, No. (%)			*P* value
	Overall (N = 398 972)	Obesity (n = 93 357)	No obesity (n = 305 615)	
Social isolation index				
0	212 204 (53.19)	45 673 (48.92)	166 531 (54.49)	<.001
1	151 544 (37.98)	37881 (40.58)	113 663 (37.19)	
≥2	35 224 (8.83)	9803 (10.5)	25 421 (8.32)	
Loneliness index				
0	271 828 (68.13)	59 342 (63.56)	212 486 (69.53)	<.001
1	102 803 (25.77)	26 627 (28.52)	76 176 (24.93)	
2	24 341 (6.1)	7388 (7.91)	16 953 (5.55)	
Age, mean (SD), y	55.85 (8.08)	56.07 (7.89)	55.79 (8.14)	<.001
Sex				
Female	220 469 (55.26)	50 139 (53.71)	170 330 (55.73)	<.001
Male	178 503 (44.74)	43 218 (46.29)	135 285 (44.27)	
Race and ethnicity				
Asian	13 734 (3.44)	2884 (3.09)	10 850 (3.55)	<.001
Black	2009 (0.5)	409 (0.44)	1600 (0.52)	
Chinese	1079 (0.27)	60 (0.06)	1019 (0.33)	
Multiracial	14 179 (3.55)	3874 (4.15)	10 305 (3.37)	
White	363 685 (91.16)	84 958 (91)	278 727 (91.2)	
Other^a^	3173 (0.8)	859 (0.92)	2314 (0.76)	
Missing categories	1113 (0.28)	313 (0.34)	800 (0.26)	
Townsend deprivation index, mean (SD)	−1.41 (3.02)	−0.97 (3.19)	−1.55 (2.96)	<.001
Education, y				
<15	150 601 (37.75)	39 890 (42.73)	110 711 (36.23)	<.001
15-19	110 084 (27.59)	28 352 (30.37)	81 732 (26.74)	
≥20	135 475 (33.96)	24 291 (26.02)	111 184 (36.38)	
Smoking status				
Never	223 236 (55.95)	49 173 (52.67)	174 063 (56.95)	<.001
Previous	133 584 (33.48)	34 937 (37.42)	98 647 (32.28)	
Current	41 042 (10.29)	8903 (9.54)	32 139 (10.52)	
Alcohol intake, times/wk				
<3	221 428 (55.5)	60 249 (64.54)	161 179 (52.74)	<.001
≥3	177 353 (44.45)	33 051 (35.4)	144 302 (47.22)	
Healthy diet score				
<3	128 836 (32.29)	33 843 (36.25)	94 993 (31.08)	<.001
≥3	257 400 (64.52)	55 692 (59.65)	201 708 (66)	
Physical activity, min/wk				
<150	100 645 (25.23)	25 682 (27.51)	74 963 (24.53)	<.001
≥150	220 906 (55.37)	43 109 (46.18)	177 797 (58.18)	
Depression	23 367 (5.86)	7346 (7.87)	16 021 (5.24)	<.001
Anxiety	6699 (1.68)	1774 (1.9)	4925 (1.61)	<.001
Eating disorder	2145 (0.54)	368 (0.39)	1777 (0.58)	<.001
Hypertension	211 801 (53.09)	66 051 (70.75)	145 750 (47.69)	<.001
High cholesterol	211 801 (53.09)	21 258 (22.77)	36 455 (11.93)	<.001
Diabetes	17 291 (4.33)	9054 (9.7)	8237 (2.7)	<.001
Metformin use	669 (0.17)	320 (0.34)	349 (0.11)	<.001
Glucocorticoid use	5690 (1.43)	1199 (1.28)	4491 (1.47)	<.001
HbA_1c_ level, mean (SD), mmol/mol	35.75 (6.31)	37.96 (8.44)	35.07 (5.33)	<.001

Abbreviation: HbA_1c_, hemoglobin A_1c_.

^a^ UK Biobank did not define other racial and ethnic groups.

### Social Isolation and Loneliness With Risk of Mortality Among People With and Without Obesity

During a median (IQR) follow-up of 12.73 (12.01-13.43) years, a total of 22 872 incident deaths were recorded, including 11 442 cancer-related deaths, 4372 CVD-related deaths, and 7058 other deaths. The unadjusted model and basic model adjusted for age and sex indicated that lower index of social isolation was significantly associated with lower risks of all-cause mortality in people with obesity (Table 2). In the multivariable adjusted model, compared with participants with obesity with the highest social isolation index (≥2), HRs for all-cause mortality were 0.85 (95% CI, 0.79- 0.91) and 0.74 (95% CI, 0.69-0.80) for participants with obesity and a social isolation index of 1 and 0, respectively (*P* for trend < .001) (Table 2). Similar results were observed for cancer-related and CVD-related mortality (eTable 5 and eTable 6 in Supplement 1). For loneliness, compared with participants with obesity with the highest loneliness index (2), HRs for all-cause mortality were 0.97 (95% CI, 0.89-1.06) and 0.86 (95% CI, 0.79-0.94) for participants with obesity and a loneliness index of 1 and 0, respectively (*P* for trend < .001) (Table 2). Similar results were observed in people without obesity (Table 3). Pairwise comparisons appear in eTable 7 and eTable 8 in Supplement 1. We found that the association of social isolation with all-cause mortality was stronger in people without obesity (*P* for interaction = .003). However, we observed that the cumulative risk of all-cause mortality across the 3 groups of social isolation in participants with obesity was still higher than that of participants without obesity and the same social isolation or loneliness index (eFigure 3 in Supplement 1). Additionally, we found the association of obesity with all-cause mortality was strengthened by social isolation index (eFigure 4 in Supplement 1).

**Table 2.** Associations of Social Isolation and Loneliness With Risk for All-Cause Mortality in 93 357 People With Obesity

Outcomes	HR (95% CI)			*P* value for trend
	Index ≥2	Index = 1	Index = 0	
**Social isolation**				
Total population				
Cases, No./person-years	1003/120 599	2900/471 488	2921/571 821	NA
Unadjusted model	1 [Reference]	0.74 (0.68-0.79)	0.61 (0.57-0.65)	<.001
Basic model^a^	1 [Reference]	0.74 (0.69-0.79)	0.58 (0.54-0.62)	<.001
Multivariable model^b^	1 [Reference]	0.85 (0.79-0.91)	0.74 (0.69-0.80)	<.001
Women				
Cases, No./person-years	441/66 483	1250/266 881	1136/295 960	NA
Unadjusted model	1 [Reference]	0.70 (0.63-0.78)	0.57 (0.51-0.64)	<.001
Basic model^a^	1 [Reference]	0.71 (0.64-0.79)	0.58 (0.52-0.64)	<.001
Multivariable model^b^	1 [Reference]	0.83 (0.74-0.93)	0.76 (0.68-0.85)	<.001
Men				
Cases, No./person-years	562/54 116	1650/204 606	1785/275 860	NA
Unadjusted model	1 [Reference]	0.77 (0.70-0.85)	0.62 (0.56-0.68)	<.001
Basic model^a^	1 [Reference]	0.76 (0.69-0.83)	0.58 (0.53-0.64)	<.001
Multivariable model^b^	1 [Reference]	0.86 (0.78-0.95)	0.72 (0.65-0.80)	<.001
**Loneliness**				
Total population				
Cases, No./person-years	615/91 851	2141/331 128	4068/740 926	NA
Unadjusted model	1 [Reference]	0.97 (0.88-1.06)	0.82 (0.75-0.89)	<.001
Basic model^a^	1 [Reference]	0.87 (0.80-0.95)	0.72 (0.66-0.78)	<.001
Multivariable model^b^	1 [Reference]	0.97 (0.89-1.06)	0.86 (0.79-0.94)	<.001
Women				
Cases, No./person-years	263/50 873	922/182 032	1642/396 419	NA
Unadjusted model	1 [Reference]	0.98 (0.86-1.13)	0.80 (0.70-0.91)	<.001
Basic model^a^	1 [Reference]	0.94 (0.82-1.07)	0.75 (0.66-0.86)	<.001
Multivariable model^b^	1 [Reference]	1.03 (0.90-1.18)	0.90 (0.79-1.03)	.004
Men				
Cases, No./person-years	352/40 979	1219/149 096	2426/344 508	NA
Unadjusted model	1 [Reference]	0.95 (0.84-1.07)	0.82 (0.73-0.92)	<.001
Basic model^a^	1 [Reference]	0.82 (0.73-0.93)	0.69 (0.62-0.77)	<.001
Multivariable model^b^	1 [Reference]	0.93 (0.82-1.05)	0.84 (0.75-0.94)	<.001

Abbreviations: HR, hazard ratio; NA, not applicable.

^a^ Basic model was adjusted for age and sex.

^b^ Multivariable model was adjusted for age, sex, race and ethnicity, Townsend Deprivation Index, years of education, smoking status, alcohol intake, healthy diet score, physical activity, depression, anxiety, eating disorder, hypertension, high cholesterol, diabetes, metformin use, glucocorticoid use, and hemoglobin A_1c_ level.

**Table 3.** Associations of Social Isolation and Loneliness With Risk for All-Cause Mortality in 305 615 People Without Obesity

Outcomes	HR (95% CI)			*P* value for trend
	Index ≥2	Index = 1	Index = 0	
**Social isolation**				
Total population				
Cases, No./person-years	2195/313 509	6331/1 424 107	7522/2 097 159	NA
Unadjusted model	1 [Reference]	0.63 (0.60-0.66)	0.51 (0.48-0.53)	<.001
Basic model^a^	1 [Reference]	0.63 (0.60-0.67)	0.50 (0.47-0.52)	<.001
Multivariable model^b^	1 [Reference]	0.75 (0.72-0.79)	0.66 (0.63-0.70)	<.001
Women				
Cases, No./person-years	843/162 450	2816/802 639	3142/1 182 823	NA
Unadjusted model	1 [Reference]	0.67 (0.62-0.73)	0.51 (0.47-0.55)	<.001
Basic model^a^	1 [Reference]	0.69 (0.64-0.74)	0.53 (0.49-0.57)	<.001
Multivariable model^b^	1 [Reference]	0.81 (0.75-0.88)	0.71 (0.65-0.77)	<.001
Men				
Cases, No./person-years	1352/151 059	3515/621 467	4380/914 338	NA
Unadjusted model	1 [Reference]	0.63 (0.59-0.67)	0.53 (0.50-0.56)	<.001
Basic model^a^	1 [Reference]	0.60 (0.56-0.64)	0.48 (0.45-0.51)	<.001
Multivariable model^b^	1 [Reference]	0.71 (0.67-0.76)	0.64 (0.60-0.68)	<.001
**Loneliness**				
Total population				
Cases, No./person-years	1185/211 332	4662/952 966	10 201/267 0477	NA
Unadjusted model	1 [Reference]	0.87 (0.82-0.93)	0.68 (0.64-0.72)	<.001
Basic model^a^	1 [Reference]	0.80 (0.75-0.85)	0.63 (0.60-0.67)	<.001
Multivariable model^b^	1 [Reference]	0.91 (0.86-0.97)	0.81 (0.76-0.86)	<.001
Women				
Cases, No./person-years	499/111 922	1914/520 378	4388/1 515 612	NA
Unadjusted model	1 [Reference]	0.83 (0.75-0.91)	0.65 (0.59-0.71)	<.001
Basic model^a^	1 [Reference]	0.81 (0.74-0.90)	0.67 (0.61-0.73)	<.001
Multivariable model^b^	1 [Reference]	0.89 (0.81-0.98)	0.81 (0.74-0.89)	<.001
Men				
Cases, No./person-years	686/99 410	2748/432 588	5813/1 154 867	NA
Unadjusted model	1 [Reference]	0.92 (0.85-1.00)	0.73 (0.67-0.79)	<.001
Basic model^a^	1 [Reference]	0.79 (0.72-0.86)	0.61 (0.56-0.66)	<.001
Multivariable model^b^	1 [Reference]	0.93 (0.86-1.02)	0.81 (0.75-0.88)	<.001

Abbreviations: HR, hazard ratio; NA, not applicable.

^a^ Basic model was adjusted for age and sex.

^b^ Multivariable model was adjusted for age, sex, race and ethnicity, Townsend Deprivation Index, years of education, smoking status, alcohol intake, healthy diet score, physical activity, depression, anxiety, eating disorder, hypertension, high cholesterol, diabetes, metformin use, glucocorticoid use, and hemoglobin A_1c_ level.

Furthermore, the associations of individual social isolation and loneliness indicators with risk of mortality were analyzed. Almost all the individual indicators of social isolation and loneliness were significantly associated with all-cause mortality and CVD-related mortality in people with obesity (eTable 9 in Supplement 1).

### Improvement of Social Isolation and Loneliness and Obesity-Related Excess Risk of Mortality Among People With Obesity Compared With Matched Control Participants Without Obesity

The adjusted cumulative hazard curves for the probability of mortality among participants without and with obesity with social isolation or loneliness index of 0, 1, or 2 or greater are presented in the Figure and eFigure 5 in Supplement 1. To estimate the extent to which the obesity-related risk of mortality could be attenuated through controlling the social isolation and loneliness index, we evaluated the risk of mortality according to the degree of social isolation and loneliness among people with obesity vs matched control participants. Compared with people without obesity, the risks of all-cause mortality among people with obesity constantly decreased with decreasing levels of social isolation and loneliness (Table 4). The findings were consistent in the unadjusted model and multivariable adjusted model. As the index of social isolation and loneliness went from highest to lowest, the HR for all-cause mortality decreased by 36% and 9% in people with obesity compared to people without obesity using multivariable model. Similar results were observed for CVD-related mortality (eTable 10 in Supplement 1). Additionally, we found a similar pattern for all-cause mortality among those with more severe obesity classes (eTable 11 in Supplement 1). Moreover, the risks of all-cause mortality and CVD-related mortality among people with obesity constantly decreased with the individual indicator of social isolation and loneliness control (eTable 12 in Supplement 1).

**Figure.**
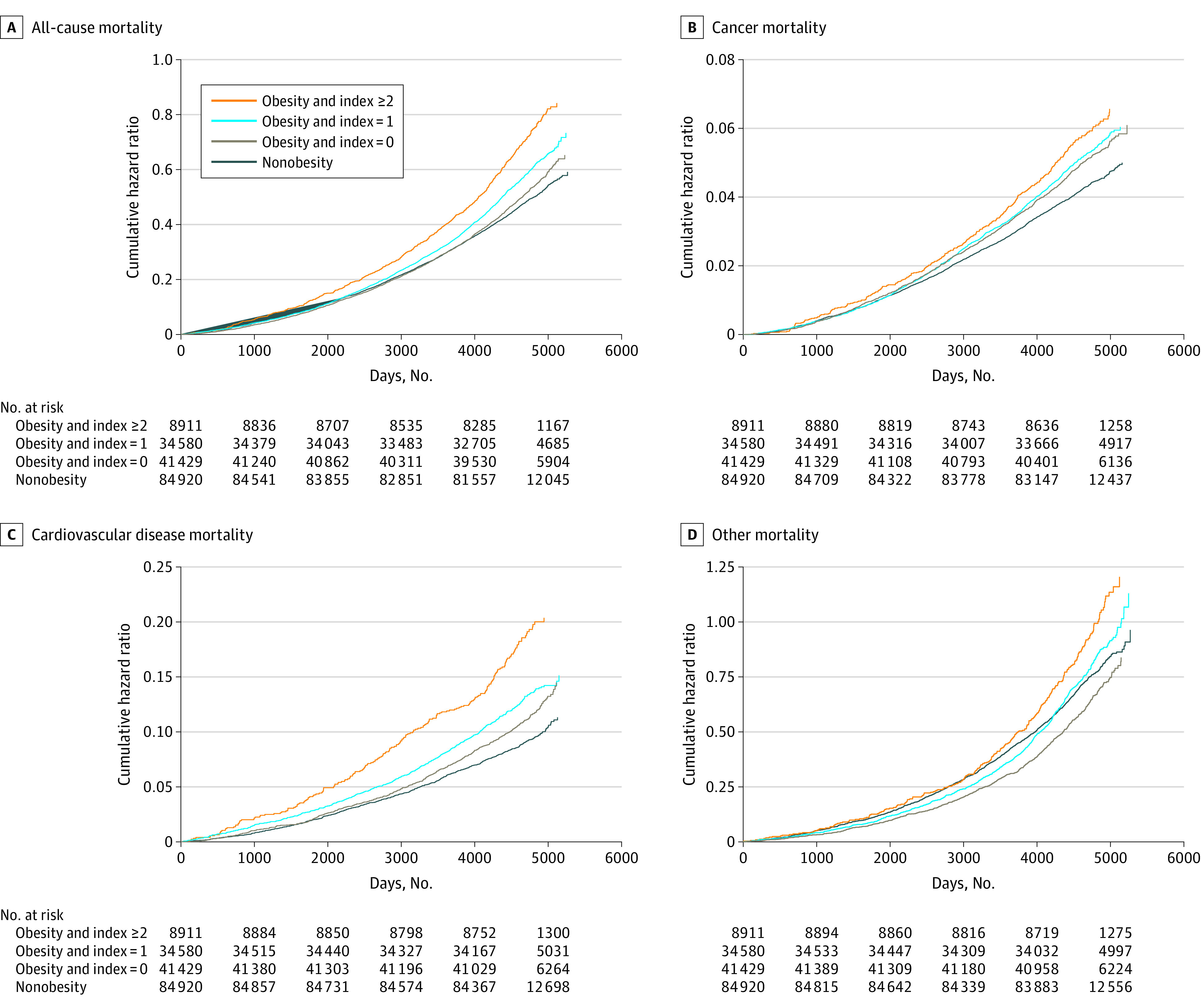
Cumulative Hazard of All-Cause and Cause-Specific Mortality for Social Isolation Index Among 84 920 Participants With Obesity Compared With 84 920 Participants Without Obesity Adjusted for age, sex, race and ethnicity, Townsend Deprivation Index, years of education, smoking status, alcohol intake, healthy diet score, physical activity, depression, anxiety, eating disorder, hypertension, high cholesterol, diabetes, metformin use, glucocorticoid use, and hemoglobin A_1c_ level.

**Table 4.** Associations of Social Isolation and Loneliness With Risk for All-Cause Mortality in 84 920 People With Obesity Compared With 84 920 Matched Control Participants Without Obesity

Outcomes	HR (95% CI)			
	No obesity	Obesity		
		Index ≥2	Index = 1	Index = 0
**Social isolation**				
Total population				
Cases, No./person-years	4398/1 064 755	876/109 740	2567/430 515	2597/518 494
Unadjusted model	1 [Reference]	2.02 (1.80-2.28)	1.58 (1.48-1.68)	1.13 (1.07-1.20)
Basic model^a^	1 [Reference]	2.03 (1.80-2.28)	1.58 (1.48-1.68)	1.13 (1.06-1.19)
Multivariable model^b^	1 [Reference]	1.37 (1.20-1.56)	1.27 (1.18-1.36)	1.01 (0.95-1.08)
Women				
Cases, No./person-years	1957/615 790	416/64 527	1205/259 888	1108/288 688
Unadjusted model	1 [Reference]	2.13 (1.79-2.54)	1.55 (1.41-1.69)	1.15 (1.05-1.25)
Basic model^a^	1 [Reference]	2.14 (1.80-2.55)	1.55 (1.41-1.70)	1.15 (1.05-1.25)
Multivariable model^b^	1 [Reference]	1.48 (1.22-1.79)	1.27 (1.14-1.41)	1.04 (0.94-1.14)
Men				
Cases, No./person-years	2441/448 964	460/45 213	1362/170 628	1489/229 806
Unadjusted model	1 [Reference]	1.93 (1.64-2.27)	1.60 (1.47-1.75)	1.11 (1.03-1.20)
Basic model^a^	1 [Reference]	1.93 (1.64-2.27)	1.61 (1.47-1.76)	1.11 (1.03-1.20)
Multivariable model^b^	1 [Reference]	1.29 (1.07-1.55)	1.27 (1.15-1.41)	0.99 (0.91-1.08)
**Loneliness**				
Total population				
Cases, No./person-years	4398/106 4755	535/83 959	1888/301 515	3617/673 274
Unadjusted model	1 [Reference]	1.68 (1.45-1.93)	1.51 (1.40-1.62)	1.30 (1.23-1.36)
Basic model^a^	1 [Reference]	1.69 (1.47-1.95)	1.50 (1.40-1.62)	1.30 (1.23-1.36)
Multivariable model^b^	1 [Reference]	1.21 (1.03-1.41)	1.18 (1.08-1.28)	1.12 (1.05-1.19)
Women				
Cases, No./person-years	1957/615 790	255/49 693	883/177 070	1591/386 341
Unadjusted model	1 [Reference]	1.70 (1.39-2.09)	1.61 (1.45-1.80)	1.28 (1.19-1.38)
Basic model^a^	1 [Reference]	1.70 (1.39-2.09)	1.61 (1.45-1.80)	1.28 (1.19-1.38)
Multivariable model^b^	1 [Reference]	1.29 (1.03-1.61)	1.30 (1.15-1.47)	1.10 (1.01-1.20)
Men				
Cases, No./person-years	2441/448 964	280/34 266	1005/124 446	2026/286 934
Unadjusted model	1 [Reference]	1.65 (1.36-2.01)	1.42 (1.29-1.57)	1.31 (1.22-1.40)
Basic model^a^	1 [Reference]	1.68 (1.38-2.04)	1.42 (1.28-1.56)	1.31 (1.22-1.40)
Multivariable model^b^	1 [Reference]	1.14 (0.92-1.42)	1.08 (0.97-1.22)	1.13 (1.04-1.23)

Abbreviation: HR, hazard ratio.

^a^ Basic model was adjusted for age and sex.

^b^ Multivariable model was adjusted for age, sex, race and ethnicity, Townsend Deprivation Index, years of education, smoking status, alcohol intake, healthy diet score, physical activity, depression, anxiety, eating disorder, hypertension, high cholesterol, diabetes, metformin use, glucocorticoid use, and hemoglobin A_1c_.

### Relative Importance of Risk Factors for Mortality in People With Obesity

Relative importance analysis indicated that social isolation ranked fourth, fourth, third, and eighth in relative strength for risk of all-cause mortality, cancer-related mortality, CVD-related mortality, and other mortality, respectively, while loneliness was in fourteenth place for risk of all-cause mortality in participants with obesity. Social isolation was ranked higher than loneliness, depression, anxiety, and lifestyle-related risk factors including alcohol, physical activity, and healthy diet (eFigure 6 in Supplement 1).

### Sensitivity Analysis

The associations of isolation and loneliness with risk of mortality did not change significantly when we excluded participants who died during the first 2 years of follow-up (eTables 13 and 14 in Supplement 1). Additionally, when all the missing covariates were inputted using multiple imputation, the results remained stable (eTables 15 and 16 in Supplement 1).

## Discussion

In this prospective cohort study with a median follow-up of 12.73 years, we found that improvement of social isolation and loneliness were associated with lower risks of all-cause mortality. Additionally, the obesity-related excess risks of all-cause mortality decreased with decreased indexes of social isolation and loneliness. Furthermore, social isolation was ranked as the fourth strongest factor, while loneliness was ranked the fourteenth, in risk of all-cause mortality compared with other lifestyle-related risk factors.

For the first time to our knowledge, our study found significant associations of social isolation and loneliness with all-cause mortality in people with obesity. Our findings are supported by several previous studies conducted in the general populations, in which positive correlations of social isolation and loneliness with mortality were observed.^31,32^ A cohort study from Finland showed that social isolation was related to a 26% increased risk of all-cause mortality in the general population when separately adjusting for socioeconomic factors, biological factors, depressive symptoms, cognitive performance, health-related behaviors, and self-rated health.^12^ Another study using data from the Swedish Panel Study of Living Conditions of the Oldest Old (SWEOLD) Study indicated that social isolation and loneliness were associated with an increased all-cause mortality risk.^16^

Obesity, social isolation, and loneliness are all associated with many health issues.^33,34^ A lack of social support may exacerbate the health-risk behaviors of people with obesity including smoking, inactivity, and unhealthy diets and might also neglect health-protective behaviors, such as adherence to medical recommendations.^35^ Moreover, those who live alone or lack social contacts may be at a heightened risk of death if they develop acute symptoms because they might not have a strong network of confidantes to urge them to seek medical attention.^36^ Addressing social isolation and loneliness in individuals with obesity may potentially help improve unhealthy lifestyles, provide better psychological support, and encourage people at high risk to seek medical assistance when necessary.

It is not surprising that the protective association between improvement of social isolation and all-cause mortality was stronger in people without obesity than those with obesity. The reason why the association between social isolation and mortality is relatively weak among people with obesity may be partly because obesity introduces various biological complications that can increase mortality risk, such as cardiovascular problems, type 2 diabetes, and chronic inflammation. These health issues closely related to obesity might mask or override the protective effects of improvement of social isolation on mortality in the obese population, making the association less pronounced compared with individuals without obesity. Importantly, our findings emphasize that more intensive interventions are needed to improve social isolation in people with obesity than people without obesity to lower the risk of mortality.

We observed that social isolation was significantly associated with CVD-related mortality in people with obesity, which may be attributed to the following reasons. CVDs are the leading cause of death globally,^37^ with relatively high incidence and mortality rates. The association of social isolation with CVD-related mortality is pronounced due to a larger population affected. Additionally, social connections and social support play an important role in heart health. Social interaction and support may reduce stress, promote healthy behaviors, provide emotional support,^38^ and reduce the risk of CVD through positive social engagement.^39^ Social isolation may weaken these factors, leading to an increased risk of CVD. Additionally, we compared the relative importance of social isolation and loneliness to depression, anxiety, the Townsend Index, and lifestyle-related risk factors in risk of mortality among people with obesity and found that social isolation ranked higher than loneliness, depression, anxiety, and lifestyle-related risk factors, including alcohol, physical activity, and healthy diet, which indicated that social isolation played a significant role in estimating the risk of mortality.

Our study indicated that social isolation was more strongly associated with mortality than loneliness, consistent with a previous study in a general population.^12^ Social isolation and loneliness are distinct factors that correlate differently with health outcomes and mortality. Social isolation measures the scarcity of contact with others and related health resources, while loneliness reflects a sense of detachment potentially linked to emotional states like depression.^12^ Individuals can experience loneliness even when married or living with others.^40^ Our results suggest that improvement of social isolation may provide more benefits for reducing risks of all-cause mortality and CVD mortality than loneliness in people with obesity.

In addition, we analyzed associations of social isolation and loneliness improvement with obesity-related excess mortality, by comparing with matched individuals without obesity. Interestingly, we found that obesity-related excess risk of all-cause mortality and CVD-related mortality could be attenuated by controlling social isolation or loneliness. The risk of all-cause mortality among people with obesity constantly decreased with decreasing levels of social isolation or loneliness. Improvement of social isolation in people with obesity could provide more benefits for reducing obesity-related risk of mortality than loneliness.

### Strengths and Limitations

This study has several strengths, including a prospective study design, a large sample size of people with obesity with data on social isolation and loneliness, and comprehensive information on covariates. However, there are also several limitations to consider. First, no data of duration of loneliness or social isolation and stability can be obtained from the UK Biobank cohort. Second, we did not adjust for cognitive function due to a limited number of participants completing the test, which warrants future research. Third, the study sample included a relatively low percentage of non-White European participants, and generalization of our findings to other racial and ethnic groups requires further investigation. Fourth, the social isolation and loneliness indexes were constructed from simple questions, which might not fully capture the complex social networking and interaction phenomenon. Fifth, the observational nature of the study limited causal inference. Sixth, UK Biobank participants may have healthier behaviors, potentially limiting the generalizability of findings. However, a representative population may not be necessary for assessing exposure-disease associations.

## Conclusions

In this cohort study of UK Biobank participants, we found that improvement in social isolation and loneliness were associated with a lower risk of all-cause mortality in people with obesity. Social isolation was more important to the estimation of risk of all-cause mortality than loneliness, depression, anxiety, and lifestyle-related risk factors, including alcohol, physical activity, and healthy diet. Importantly, our results indicated that control of social isolation and loneliness might attenuate the obesity-related excess risk of all-cause mortality. Our findings lend support to social isolation and loneliness control to decrease the risk of all-cause mortality in people with obesity.

## Notes

Supplement 1. **eFigure 1.** Flowchart of Participant Enrollment **eFigure 2.** Directed Acyclic Graph (DAG) Indicating the Associations Among Exposures, Outcomes, and Included Covariates In The Analyses **eFigure 3.** Cumulative Hazard of All-Cause Mortality Among Participants With Obesity Compared With Participants Without Obesity With Different Social Isolation Index and Loneliness Index **eFigure 4.** Joint Association of Social Isolation and Obesity With All-Cause Mortality via Multivariable Model **eFigure 5.** Cumulative Hazard of All-Cause and Cause-Specific Mortality for Loneliness Index Among 84 920 Participants With Obesity Compared With 84 920 Participants Without Obesity **eFigure 6.** Relative Importance of Risk Factors for Mortality in 93 357 People With Obesity **eTable 1.** Evaluation of Social Isolation and Loneliness in the UK Biobank **eTable 2.** Assessment of Healthy Diet Score in the UK Biobank **eTable 3.** Self-Reported and *ICD-10* Codes for Prevalent Diseases in This Study **eTable 4.** Summary of Medications for Metformin Use and Glucocorticoid Use **eTable 5.** Numbers and Percentages of Participants With Missing Covariates **eTable 6.** Associations of Social Isolation and Loneliness With Risk for Cancer-Related and CVD-Related Mortality in 93 357 People With Obesity **eTable 7.** Pairwise Comparisons for Associations of Social Isolation and Loneliness With Risk for All-Cause Mortality in 93 357 People With Obesity **eTable 8.** Pairwise Comparisons for Associations of Social Isolation and Loneliness With Risk for All-Cause Mortality in 305 615 People Without Obesity **eTable 9.** Association of Individual Components of Social Isolation and Loneliness With Risk of All-Cause Mortality and Cause-Specific Mortality in 93 357 People With Obesity via Multivariable Model **eTable 10.** Associations of Social Isolation and Loneliness With Risk for Cancer-Related and CVD-Related Mortality in 84 920 People With Obesity Compared With 84 920 Matched Control Participants via Multivariable Model **eTable 11.** Associations of Social Isolation and Loneliness With Risk for All-Cause Mortality in 84 920 People With Different Obesity Classes Compared With 84 920 Matched Control Participants via Multivariable Model **eTable 12.** Association of Individual Components of Social Isolation and Loneliness With Risk of All-Cause Mortality and Cause-Specific Mortality in 84 920 People With Obesity Compared With 84 920 Matched Control Participants via Multivariable Model **eTable 13.** Associations of Social Isolation and Loneliness With Risk of All-Cause Mortality and Cause-Specific Mortality in 93 037 People With Obesity via Multivariable Model After Excluding Participants Who Died During the First 2 Years of Follow-Up **eTable 14.** Associations of Social Isolation and Loneliness With Risk of All-Cause Mortality and Cause-Specific Mortality in 84 920 People With Obesity Compared With 84 920 Matched Control Participants Multivariable Model After Excluding Participants Who Died During the First 2 Years of Follow-Up **eTable 15.** Associations of Social Isolation and Loneliness With Risk of All-Cause Mortality and Cause-Specific Mortality in 93 357 People With Obesity via Multivariable Model With All Missing Covariate Data Imputed Using Multiple Imputation **eTable 16.** Associations of Social Isolation and Loneliness With Risk of All-Cause Mortality and Cause-Specific Mortality in 84 920 People With Obesity Compared With 84 920 Matched Control Participants via Multivariable Model With All Missing Covariate Data Imputed Using Multiple Imputation

Supplement 2.
Data Sharing Statement
