# Supplementary material for: Improvement of Social Isolation and Loneliness and Excess Mortality Risk in People With Obesity
Source: JAMA Netw Open. 2024 Jan 22;7(1):e2352824. doi: 10.1001/jamanetworkopen.2023.52824 (PMC10804268; doi:10.1001/jamanetworkopen.2023.52824)
Supplement: Supplement 1. — eFigure 1. Flowchart of Participant Enrollment eFigure 2. Directed Acyclic Graph (DAG) Indicating the Associations Among Exposures, Outcomes, and Included Covariates In The Analyses eFigure 3. Cumulative Hazard of All-Cause Mortality Among Participants With Obesity Compared With Participants Without Obesity With Different Social Isolation Index and Loneliness Index eFigure 4. Joint Association of Social Isolation and Obesity With All-Cause Mortality via Multivariable Model eFigure 5. Cumulative Hazard of All-Cause and Cause-Specific Mortality for Loneliness Index Among 84 920 Participants With Obesity Compared With 84 920 Participants Without Obesity eFigure 6. Relative Importance of Risk Factors for Mortality in 93 357 People With Obesity eTable 1. Evaluation of Social Isolation and Loneliness in the UK Biobank eTable 2. Assessment of Healthy Diet Score in the UK Biobank eTable 3. Self-Reported and ICD-10 Codes for Prevalent Diseases in This Study eTable 4. Summary of Medications for Metformin Use and Glucocorticoid Use eTable 5. Numbers and Percentages of Participants With Missing Covariates eTable 6. Associations of Social Isolation and Loneliness With Risk for Cancer-Related and CVD-Related Mortality in 93 357 People With Obesity eTable 7. Pairwise Comparisons for Associations of Social Isolation and Loneliness With Risk for All-Cause Mortality in 93 357 People With Obesity eTable 8. Pairwise Comparisons for Associations of Social Isolation and Loneliness With Risk for All-Cause Mortality in 305 615 People Without Obesity eTable 9. Association of Individual Components of Social Isolation and Loneliness With Risk of All-Cause Mortality and Cause-Specific Mortality in 93 357 People With Obesity via Multivariable Model eTable 10. Associations of Social Isolation and Loneliness With Risk for Cancer-Related and CVD-Related Mortality in 84 920 People With Obesity Compared With 84 920 Matched Control Participants via Multivariable Model eTable 11. Associations of Socia [file jamanetwopen-e2352824-s001.pdf]

Supplemental Online Content

Zhou J, Tang R, Wang X, Li X, Heianza Y, Qi L. Improvement of social isolation and loneliness and excess mortality risk in people with obesity. *JAMA Netw Open.* 2024;7(1):e2352824. doi:10.1001/jamanetworkopen.2023.52824

eFigure 1. Flowchart of Participant Enrollment

eFigure 2. Directed Acyclic Graph (DAG) Indicating the Associations Among Exposures, Outcomes, and Included Covariates In The Analyses

eFigure 3. Cumulative Hazard of All-Cause Mortality Among Participants With Obesity Compared With participants Without Obesity With Different Social Isolation Index and Loneliness Index

eFigure 4. Joint Association of Social Isolation and Obesity With All-Cause Mortality via Multivariable Model

eFigure 5. Cumulative Hazard of All-Cause and Cause-Specific Mortality for Loneliness Index Among 84 920 Participants With Obesity Compared With 84 920 Participants Without Obesity

eFigure 6. Relative Importance of Risk Factors for Mortality in 93 357 People With Obesity

eTable 1. Evaluation of Social Isolation and Loneliness in the UK Biobank

eTable 2. Assessment of Healthy Diet Score in the UK Biobank

eTable 3. Self-reported and *ICD-10* Codes for Prevalent Diseases in This Study

eTable 4. Summary of Medications for Metformin Use and Glucocorticoid Use

eTable 5. Numbers and Percentages of Participants With Missing Covariates

eTable 6. Associations of Social Isolation and Loneliness With Risk for Cancer-Related and CVD-Related Mortality in 93 357 People With Obesity

eTable 7. Pairwise Comparisons for Associations of Social Isolation and Loneliness With Risk for All-Cause Mortality in 93 357 People With Obesity

eTable 8. Pairwise Comparisons for Associations of Social Isolation and Loneliness With Risk for All-Cause Mortality in 305 615 People Without Obesity

eTable 9. Association of Individual Components of Social Isolation and Loneliness With Risk of All-Cause Mortality and Cause-Specific Mortality in 93 357 People With Obesity via Multivariable Model

eTable 10. Associations of Social Isolation and Loneliness With Risk for Cancer-Related and CVD-Related Mortality in 84 920 People With Obesity Compared With 84 920 Matched Control Participants via Multivariable Model

© 2024 Zhou J et al. *JAMA Network Open.*

eTable 11. Associations of Social Isolation and Loneliness With Risk for All-Cause Mortality in 84 920 People With Different Obesity Classes Compared With 84 920 Matched Control Participants via Multivariable Model

eTable 12. Association of Individual Components of Social Isolation and Loneliness With Risk of All-Cause Mortality and Cause-Specific Mortality in 84 920 People With Obesity Compared With 84 920 Matched Control Participants via Multivariable Model

eTable 13. Associations of Social Isolation and Loneliness With Risk of All-Cause Mortality and Cause-Specific Mortality in 93 037 People With Obesity via Multivariable Model After Excluding Participants Who Died During the First 2 Years of Follow-Up

eTable 14. Associations of Social Isolation and Loneliness With Risk of All-Cause Mortality and Cause-Specific Mortality in 84 920 People With Obesity Compared With 84 920 Matched Control Participants Multivariable Model After Excluding Participants Who Died During the First 2 Years of Follow-Up

eTable 15. Associations of Social Isolation and Loneliness With Risk of All-Cause Mortality and Cause-Specific Mortality in 93 357 People With Obesity via Multivariable Model With All Missing Covariate Data Imputed Using Multiple Imputation

eTable 16. Associations of Social Isolation and Loneliness With Risk of All-Cause Mortality and Cause-Specific Mortality in 84 920 People With Obesity Compared With 84 920 Matched Control Participants via Multivariable Model With All Missing Covariate Data Imputed Using Multiple Imputation

This supplemental material has been provided by the authors to give readers additional information about their work.

© 2024 Zhou J et al. *JAMA Network Open.*

eFigure 1. Flowchart of Participant Enrollment

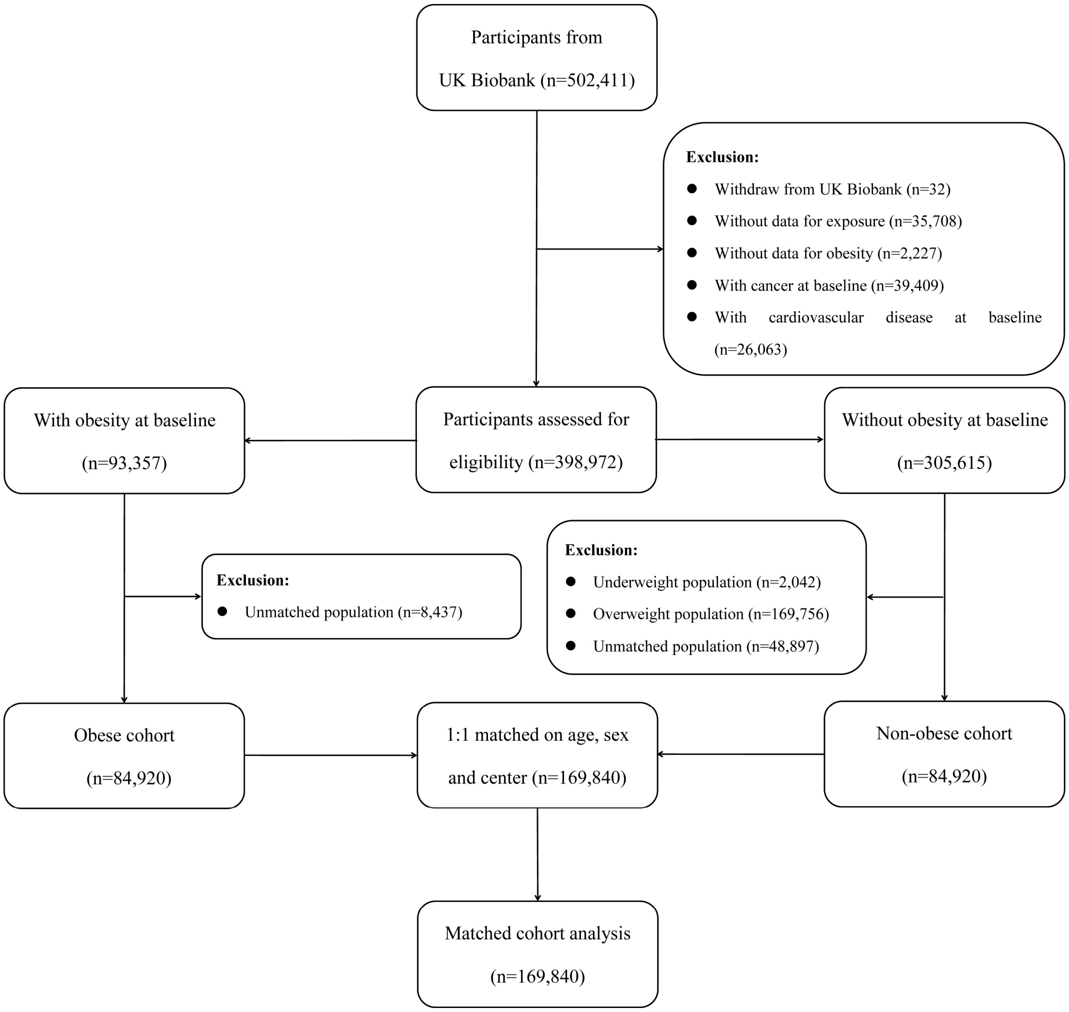

© 2024 Zhou J et al. *JAMA Network Open.*

eFigure 2. Directed Acyclic Graph (DAG) Indicating the Associations Among Exposures, Outcomes, and Included Covariates In The Analyses

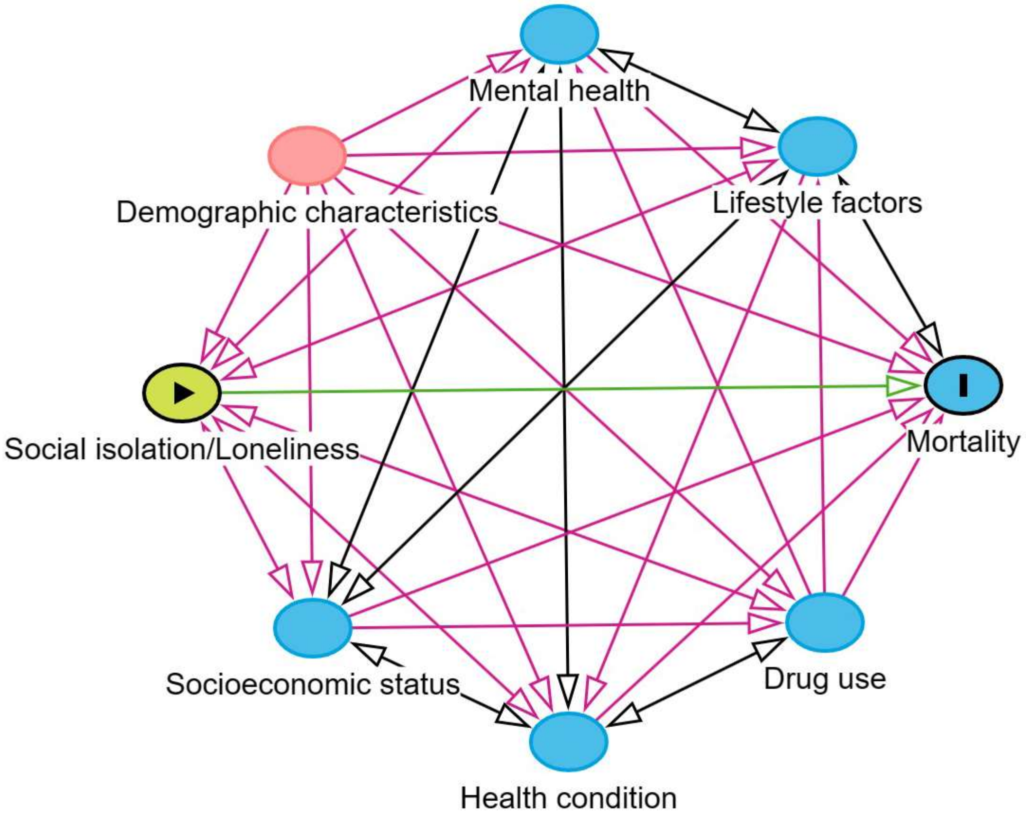

Demographic characteristics (age, sex, ethnic background) may act as confounders; Socioeconomic status (Townsend Deprivation Index and education years), lifestyle factors (smoking status, alcohol intake, healthy diet score, physical activity), Mental health (depression, anxiety, eating disorder), health condition (hypertension, high cholesterol, diabetes, HbA1c) and drug use (metformin use, glucocorticoid use) may act as confounders or mediators or both in the association between social isolation/loneliness and mortality risk.
DAG was drawn using http://www.dagitty.net/.

© 2024 Zhou J et al. *JAMA Network Open.*

eFigure 3. Cumulative Hazard of All-Cause Mortality Among Participants With Obesity Compared With participants Without Obesity With Different Social Isolation Index and Loneliness Index

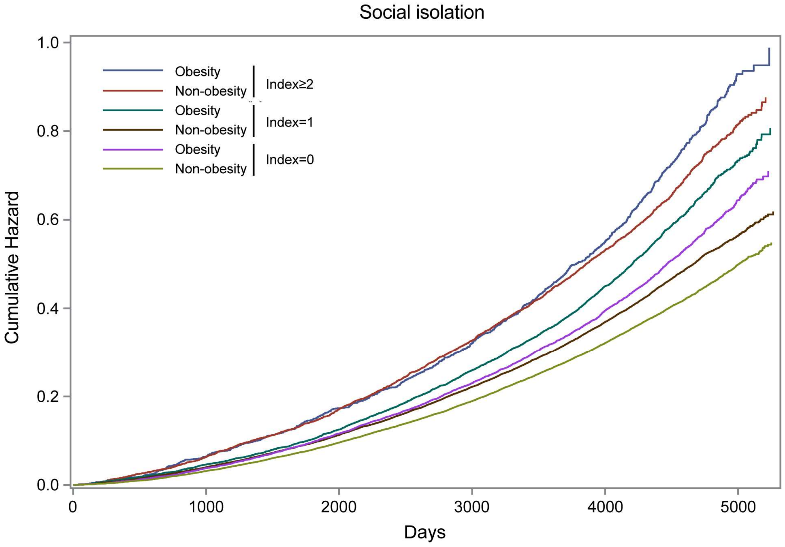

No. at risk

Index≥2	Obesity	9803	9713	9564	9368	9081	1353
	Non-obesity	25421	25208	24851	24352	23734	3507
Index=1	Obesity	37881	37654	37270	36639	35774	5387
	Non-obesity	113663	113127	112167	110757	108911	16867
Index=0	Obesity	45673	45462	45031	44416	43552	6887
	Non-obesity	166531	165966	164818	163179	160907	24705

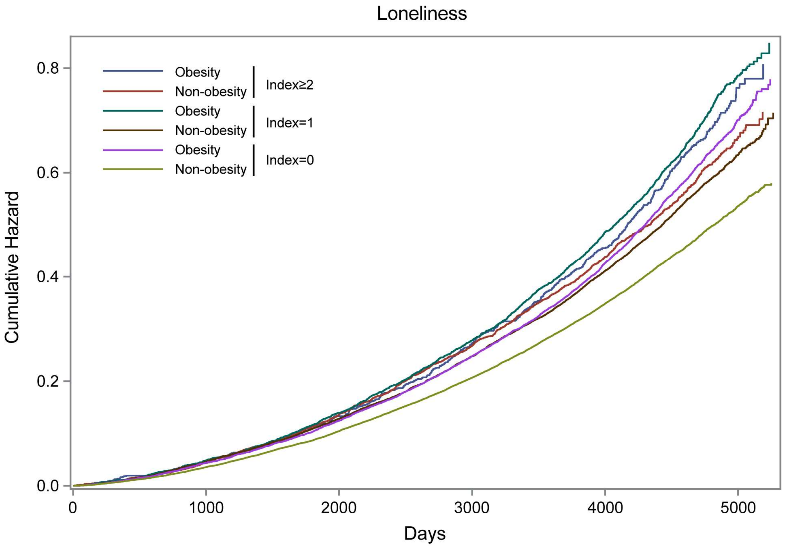

No. at risk

Index≥2	Obesity	7388	7343	7253	7118	6946	1083
	Non-obesity	16953	16858	16660	16387	16046	2493
Index=1	Obesity	26627	26460	26156	25702	25051	3916
	Non-obesity	76176	75767	75050	74030	72682	11048
Index=0	Obesity	59342	59026	58456	57603	56410	8628
	Non-obesity	212486	211676	210126	207871	204824	31538

© 2024 Zhou J et al. *JAMA Network Open.*

eFigure 4. Joint Association of Social Isolation and Obesity With All-Cause Mortality via Multivariable Model

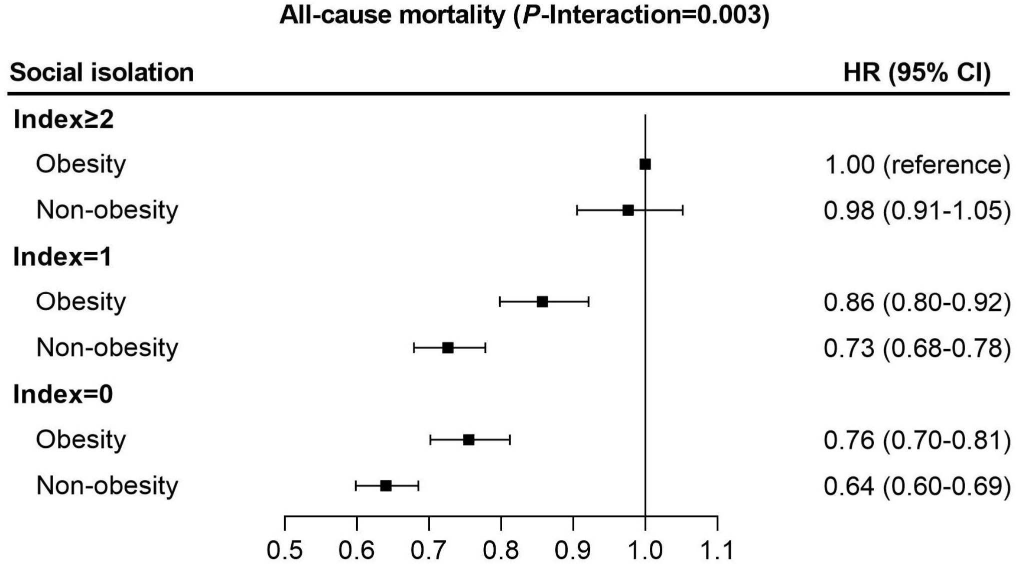

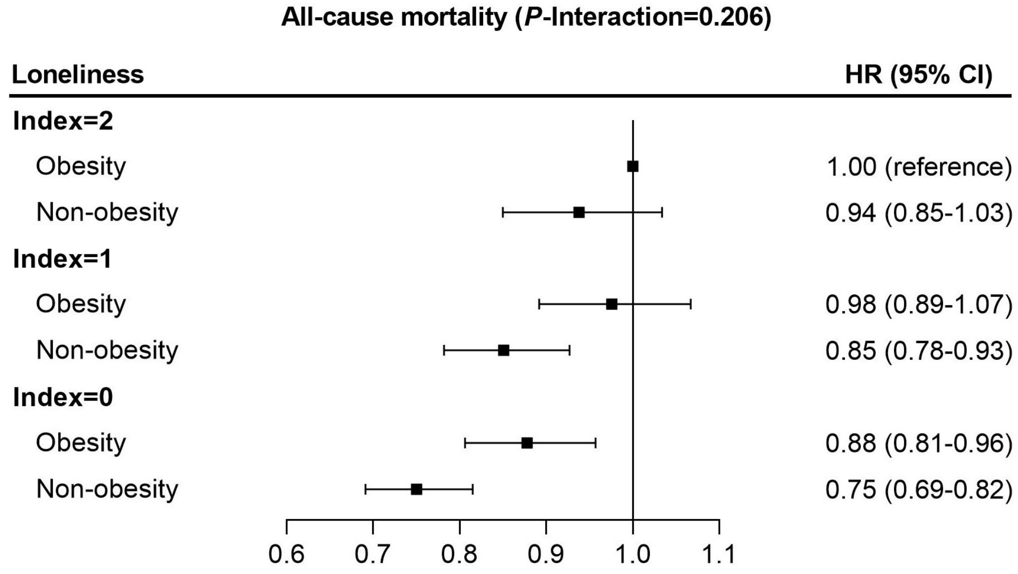

Multivariable model: adjusted for age, sex, ethnic background, Townsend Deprivation Index, education years, smoking status, alcohol intake, healthy diet score, physical activity, depression, anxiety, eating disorder, hypertension, high cholesterol, diabetes, metformin use, glucocorticoid use and HbA1c.

© 2024 Zhou J et al. *JAMA Network Open.*

eFigure 5. Cumulative Hazard of All-Cause and Cause-Specific Mortality for Loneliness Index Among 84 920 Participants With Obesity Compared With 84 920 Participants Without Obesity

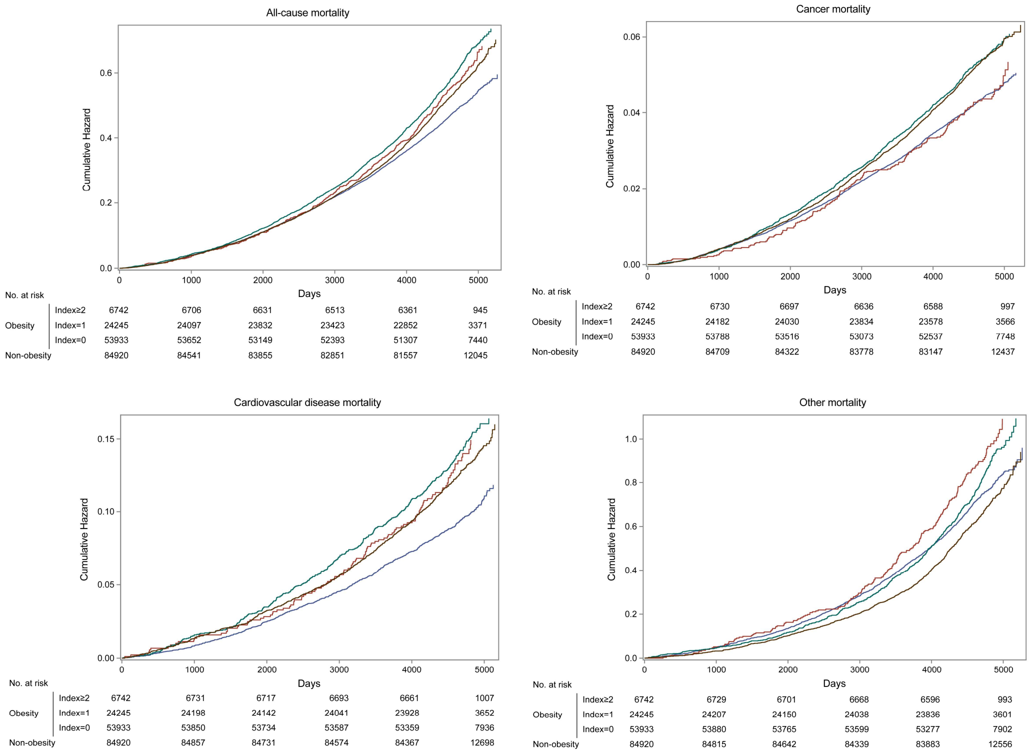

Adjusted for age, sex, ethnic background, Townsend Deprivation Index, education years, smoking status, alcohol intake, healthy diet score, physical activity, depression, anxiety, eating disorder, hypertension, high cholesterol, diabetes, metformin use, glucocorticoid use and HbA1c.

© 2024 Zhou J et al. *JAMA Network Open.*

eFigure 6. Relative Importance of Risk Factors for Mortality in 93 357 People With Obesity

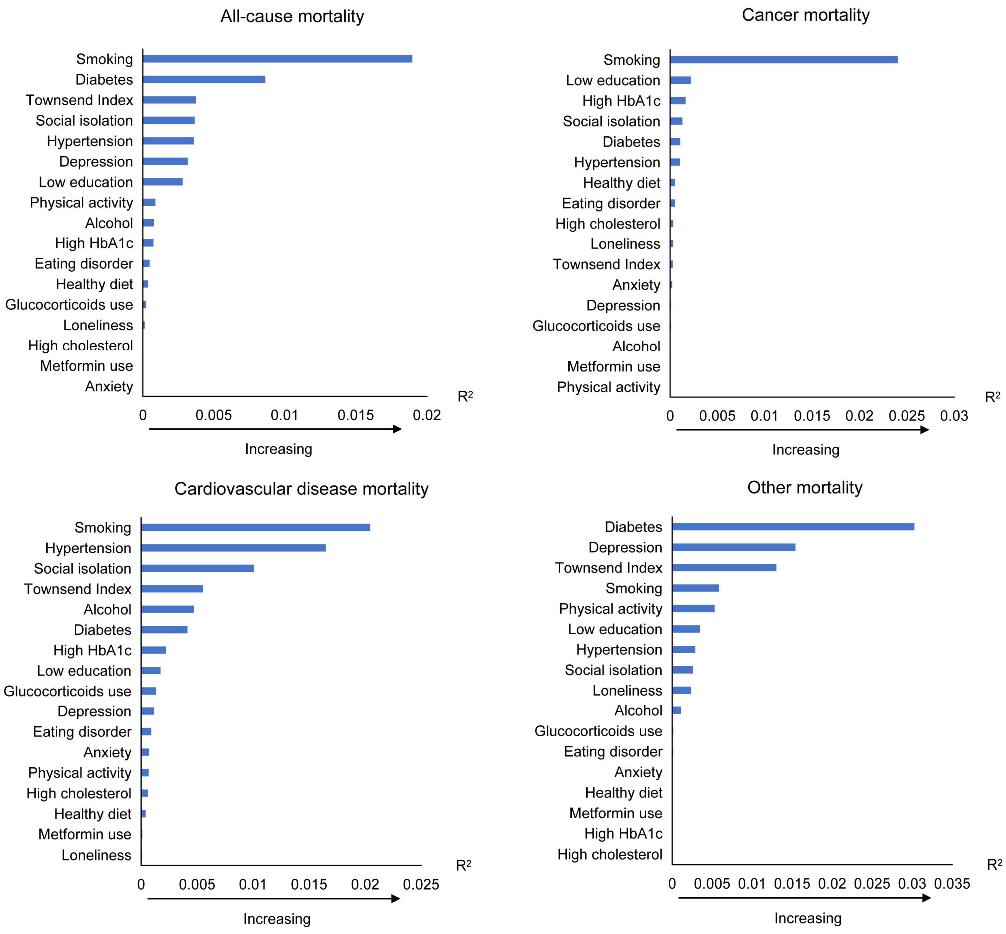

R^2 was generated by developed applications for the multivariable model: adjusted for age, sex, ethnic background, Townsend Deprivation Index, education years, smoking status, alcohol intake, healthy diet score, physical activity, depression, anxiety, eating disorder, hypertension, high cholesterol, diabetes, metformin use, glucocorticoid use and HbA1c.

© 2024 Zhou J et al. *JAMA Network Open.*

eTable 1. Evaluation of Social Isolation and Loneliness in the UK Biobank

Exposures	UK Biobank field ID	ACE touchscreen questions	Index	Responses
Social isolation indicators	709	Including yourself, how many people are living together in your household?	0	● Not living alone.
			1	● Living alone
	1031	How often do you visit friends or family or have them visit you?	0	● Almost daily<br>● 2-4 times a week<br>● About once a week<br>● About once a month
			1	● Once every few months<br>● Never or almost never<br>● No friends/family outside household
	6160	Which of the following (sports club or gym, pub or social club, religious group, adult education class, other group activity) do you attend once a week or more often?	0	● Sports club or gym<br>● Pub or social club<br>● Religious group<br>● Adult education class<br>● Other group activity
			1	● None of the above
Loneliness indicators	2020	Do you often feel lonely?	0	● No
			1	● Yes
	2110	How often are you able to confide in someone close to you?	0	● Almost daily<br>● 2-4 times a week<br>● About once a week<br>● About once a month
			1	● Once every few months<br>● Never or almost never

© 2024 Zhou J et al. *JAMA Network Open.*

eTable 2. Assessment of Healthy Diet Score in the UK Biobank

Diet	UK Biobank field ID	Description	Healthy diet score
Vegetable	1289	Cooked vegetable intake	1 for ≥4 tablespoons/day
	1299	Salad/raw vegetable intake	0 for <4 tablespoons/day
Fruit	1309	Fresh fruit intake	1 for ≥3 pieces/day
	1319	Dried fruit intake	0 for <3 pieces/day
Fish	1329	Oily fish intake	1 for ≥2 times/week
	1339	Non-oily fish intake	0 for <2 times/week
Processed meat	1349	Processed meat intake	1 for <2 times/week<br>0 for ≥2 times/week
Unprocessed red meat	1369	Beef intake	1 for <2 times/week<br>0 for ≥2 times/week
	1379	Lamb/mutton intake	
	1389	Pork intake	

© 2024 Zhou J et al. *JAMA Network Open.*

eTable 3. Self-reported and *ICD-10* Codes for Prevalent Diseases in This Study

**Prevalent diseases**	**Self reported codes**	**ICD10 codes**
Cancer	2453 (1)	C00-C97
Cardiovascular disease	6150 (1, 2, 3)	I20-I25, I60-I64
Hypertension	6150 (4)	I10
Diabetes	2443 (1)	E10-E14
High cholesterol	20002 (1473)	E78
Depression	20002 (1286)	F32
Anxiety	20002 (1287)	F06, F34, F40, F41, F42, F43, F48, F93, F99, R45
Eating disorder	20002 (1470)	R63

© 2024 Zhou J et al. *JAMA Network Open.*

eTable 4. Summary of Medications for Metformin Use and Glucocorticoid Use

Medications	Meanings	Codes
Metformin use	metformin	1140884600
	rosiglitazone 1mg / metformin 500mg tablet	1141189090
Glucocorticoid use	prednisone	1140868364
	prednisolone	1140874930
	prednisolone product	1141157402
	methylprednisolone	1140874976
	budesonide	1140862572
	budesonide product	1141157418
	novolizer budesonide 200micrograms/dose cartridge+inhaler	1141195280
	respiratory mometasone	1141191748
	mometasone	1140888172
	fluticasone	1140888098
	salmeterol+fluticasone propionate	1141164086
	rino clenil 50micrograms nasal spray	1141192916
	beclomethasone	1140884654
	beclomethasone dipropionate+salbutamol	1140881938
	pulvinal beclomethasone diprop 100mcg breath-act dry pdr inh	1141179072
	triamcinolone	1140868426
	syntaris 25micrograms nasal spray	1140876052
	flixonase 50micrograms aqueous nasal spray	1140876076
	beconase 50micrograms nasal spray	1140876136
	rhinocort 50micrograms nasal spray	1140876146
	dexa-rhinaspray nasal spray	1140876164
	zonivent aquanasal 50micrograms spray	1140928074
	nasobec aqueous 50micrograms nasal spray	1141145638
	nasacort 55micrograms aqueous nasal spray	1141146508
	nasonex 0.05% aqueous nasal spray	1141150944
	beclo-aqua 50 nasal spray	1141163146
	beclomist 50micrograms nasal spray	1141167708
	vivabec 50micrograms nasal spray	1141184608
	dexa-rhinaspray duo aqueous nasal spray	1141166848
	pollenase 50micrograms nasal spray	1141188502
	care hayfever relief 50micrograms nasal spray	1141179954

© 2024 Zhou J et al. *JAMA Network Open.*

eTable 5. Numbers and Percentages of Participants With Missing Covariates

Variable	Overall	Non-obesity	Obesity
	N (%)	N (%)	N (%)
Physical activity	77421 (19.41)	52855 (17.29)	24566 (26.31)
HbA1c	24248 (6.08)	18115 (5.93)	6133 (6.57)
Healthy diet score	12736 (3.19)	8914 (2.92)	3822 (4.09)
Education	2812 (0.7)	1988 (0.65)	824 (0.88)
Ethnic background	1113 (0.28)	800 (0.26)	313 (0.34)
Smoking status	1110 (0.28)	766 (0.25)	344 (0.37)
Townsend Deprivation Index	502 (0.13)	363 (0.12)	139 (0.15)
Alcohol intake	191 (0.05)	134 (0.04)	57 (0.06)

© 2024 Zhou J et al. *JAMA Network Open.*

eTable 6. Associations of Social Isolation and Loneliness With Risk for Cancer-Related and CVD-Related Mortality in 93 357 People With Obesity

Outcomes	Obesity			
	**Index≥2**	**Index=1**	**Index=0**	***P*-trend**
**Social isolation**				
**Cancer mortality**				
Cases/person-years	402/123278	1308/478205	1470/577824	
Unadjusted model	1.00 (reference)	0.84 (0.75-0.94)	0.78 (0.70-0.87)	<0.001
Basic model	1.00 (reference)	0.84 (0.75-0.94)	0.75 (0.67-0.84)	<0.001
Multivariable model	1.00 (reference)	0.91 (0.81-1.02)	0.86 (0.77-0.97)	0.011
**CVD mortality**				
Cases/person-years	263/123886	636/481369	617/582196	
Unadjusted model	1.00 (reference)	0.62 (0.54-0.72)	0.50 (0.43-0.57)	<0.001
Basic model	1.00 (reference)	0.62 (0.54-0.72)	0.47 (0.40-0.54)	<0.001
Multivariable model	1.00 (reference)	0.74 (0.64-0.85)	0.63 (0.54-0.74)	<0.001
**Loneliness**				
**Cancer mortality**				
Cases/person-years	228/93538	941/336253	2011/749515	
Unadjusted model	1.00 (reference)	1.15 (0.99-1.33)	1.10 (0.96-1.26)	0.688
Basic model	1.00 (reference)	1.05 (0.91-1.21)	0.98 (0.85-1.12)	0.212
Multivariable model	1.00 (reference)	1.11 (0.96-1.28)	1.07 (0.93-1.23)	0.835
**CVD mortality**				
Cases/person-years	144/93923	489/338516	883/755011	
Unadjusted model	1.00 (reference)	0.94 (0.78-1.14)	0.76 (0.64-0.91)	<0.001
Basic model	1.00 (reference)	0.85 (0.71-1.02)	0.67 (0.56-0.79)	<0.001
Multivariable model	1.00 (reference)	0.97 (0.80-1.17)	0.84 (0.70-1.01)	0.008

CVD: cardiovascular disease.

Basic model: adjusted for age and sex.

Multivariable model: adjusted for age, sex, ethnic background, Townsend Deprivation Index, education years, smoking status, alcohol intake, healthy diet score, physical activity, depression, anxiety, eating disorder, hypertension, high cholesterol, diabetes, metformin use, glucocorticoid use and HbA1c.

eTable 7. Pairwise Comparisons for Associations of Social Isolation and Loneliness With Risk for All-Cause Mortality in 93 357 People With Obesity

**Outcomes**	**Obesity**	
	**Index=1**	**Index=0**
**Social isolation**		
**Total population**		
Cases/person-years	2900/471488	2921/571821
Unadjusted model	1.00 (reference)	0.83 (0.79-0.87)
Basic model	1.00 (reference)	0.79 (0.75-0.83)
Multivariable model	1.00 (reference)	0.87 (0.83-0.92)
**Women**		
Cases/person-years	1250/266881	1136/295960
Unadjusted model	1.00 (reference)	0.82 (0.75-0.89)
Basic model	1.00 (reference)	0.81 (0.75-0.88)
Multivariable model	1.00 (reference)	0.92 (0.84-0.99)
**Men**		
Cases/person-years	1650/204606	1785/275860
Unadjusted model	1.00 (reference)	0.80 (0.75-0.85)
Basic model	1.00 (reference)	0.76 (0.72-0.82)
Multivariable model	1.00 (reference)	0.84 (0.79-0.90)
**Loneliness**		
**Total population**		
Cases/person-years	2141/331128	4068/740926
Unadjusted model	1.00 (reference)	0.85 (0.81-0.89)
Basic model	1.00 (reference)	0.82 (0.78-0.87)
Multivariable model	1.00 (reference)	0.89 (0.85-0.94)
**Women**		
Cases/person-years	922/182032	1642/396419
Unadjusted model	1.00 (reference)	0.82 (0.75-0.89)
Basic model	1.00 (reference)	0.80 (0.74-0.87)
Multivariable model	1.00 (reference)	0.87 (0.81-0.95)
**Men**		
Cases/person-years	1219/149096	2426/344508
Unadjusted model	1.00 (reference)	0.86 (0.80-0.92)
Basic model	1.00 (reference)	0.84 (0.78-0.90)
Multivariable model	1.00 (reference)	0.90 (0.84-0.97)

Basic model: adjusted for age and sex.

Multivariable model: adjusted for age, sex, ethnic background, Townsend Deprivation Index, education years, smoking status, alcohol intake, healthy diet score, physical activity, depression, anxiety, eating disorder, hypertension, high cholesterol, diabetes, metformin use, glucocorticoid use and HbA1c.

© 2024 Zhou J et al. *JAMA Network Open.*

eTable 8. Pairwise Comparisons for Associations of Social Isolation and Loneliness With Risk for All-Cause Mortality in 305 615 People Without Obesity

Outcomes	Non-obesity	
	**Index=1**	**Index=0**
**Social isolation**		
**Total population**		
Cases/person-years	6331/1424107	7522/2097159
Unadjusted model	1.00 (reference)	0.81 (0.78-0.83)
Basic model	1.00 (reference)	0.78 (0.76-0.81)
Multivariable model	1.00 (reference)	0.89 (0.86-0.92)
**Women**		
Cases/person-years	2816/802639	3142/1182823
Unadjusted model	1.00 (reference)	0.76 (0.72-0.80)
Basic model	1.00 (reference)	0.77 (0.73-0.81)
Multivariable model	1.00 (reference)	0.87 (0.83-0.92)
**Men**		
Cases/person-years	3515/621467	4380/914338
Unadjusted model	1.00 (reference)	0.85 (0.81-0.88)
Basic model	1.00 (reference)	0.80 (0.76-0.83)
Multivariable model	1.00 (reference)	0.90 (0.86-0.94)
**Loneliness**		
**Total population**		
Cases/person-years	4662/952966	10201/2670477
Unadjusted model	1.00 (reference)	0.78 (0.75-0.81)
Basic model	1.00 (reference)	0.80 (0.77-0.82)
Multivariable model	1.00 (reference)	0.89 (0.86-0.92)
**Women**		
Cases/person-years	1914/520378	4388/1515612
Unadjusted model	1.00 (reference)	0.79 (0.75-0.83)
Basic model	1.00 (reference)	0.83 (0.78-0.87)
Multivariable model	1.00 (reference)	0.91 (0.86-0.96)
**Men**		
Cases/person-years	2748/432588	5813/1154867
Unadjusted model	1.00 (reference)	0.79 (0.76-0.83)
Basic model	1.00 (reference)	0.77 (0.74-0.81)
Multivariable model	1.00 (reference)	0.87 (0.83-0.91)

Basic model: adjusted for age and sex.

Multivariable model: adjusted for age, sex, ethnic background, Townsend Deprivation Index, education years, smoking status, alcohol intake, healthy diet score, physical activity, depression, anxiety, eating disorder, hypertension, high cholesterol, diabetes, metformin use, glucocorticoid use and HbA1c.

© 2024 Zhou J et al. *JAMA Network Open.*

eTable 9. Association of Individual Components of Social Isolation and Loneliness With Risk of All-Cause Mortality and Cause-Specific Mortality in 93 357 People With Obesity via Multivariable Model

**Indicators**	**Index=1**	**Index=0**		
		**All-cause mortality**	**Cancer mortality**	**CVD mortality**
**Social isolation indicators**				
Live alone	1 (Reference)	0.79 (0.75-0.84)	0.90 (0.83-0.98)	0.72 (0.64-0.81)
Frequency of friend/family visits	1 (Reference)	0.81 (0.75-0.87)	0.87 (0.78-0.98)	0.76 (0.65-0.88)
Leisure/social activities	1 (Reference)	0.94 (0.89-0.99)	0.98 (0.90-1.05)	0.90 (0.81-1.00)
**Loneliness indicators**				
Lonely	1 (Reference)	0.87 (0.82-0.92)	1.00 (0.92-1.09)	0.87 (0.77-0.98)
Able to confide	1 (Reference)	0.93 (0.88-0.98)	1.01 (0.93-1.10)	0.90 (0.80-1.01)

CVD: cardiovascular disease.

Multivariable model: adjusted for age, sex, ethnic background, Townsend Deprivation Index, education years, smoking status, alcohol intake, healthy diet score, physical activity, depression, anxiety, eating disorder, hypertension, high cholesterol, diabetes, metformin use, glucocorticoid use and HbA1c.

© 2024 Zhou J et al. *JAMA Network Open.*

eTable 10. Associations of Social Isolation and Loneliness With Risk for Cancer-Related and CVD-Related Mortality in 84 920 People With Obesity Compared With 84 920 Matched Control Participants via Multivariable Model

Outcomes	Non-obesity	Obesity		
		Index≥2	Index=1	Index=0
**Social isolation**				
**Cancer mortality**				
Cases/person-years	2209/1074706	358/112002	1174/436370	1311/523821
Unadjusted model	1 (Reference)	1.59 (1.34-1.88)	1.37 (1.26-1.50)	1.16 (1.07-1.26)
Basic model	1 (Reference)	1.59 (1.34-1.88)	1.37 (1.26-1.50)	1.16 (1.07-1.26)
Multivariable model	1 (Reference)	1.32 (1.10-1.59)	1.31 (1.18-1.45)	1.15 (1.05-1.26)
**CVD mortality**				
Cases/person-years	719/1082451	219/112654	541/439343	536/527748
Unadjusted model	1 (Reference)	3.09 (2.36-4.04)	2.21 (1.90-2.58)	1.34 (1.17-1.52)
Basic model	1 (Reference)	3.07 (2.34-4.02)	2.23 (1.91-2.59)	1.31 (1.15-1.49)
Multivariable model	1 (Reference)	1.80 (1.33-2.44)	1.50 (1.26-1.80)	1.09 (0.93-1.27)
**Loneliness**				
**Cancer mortality**				
Cases/person-years	2209/1074706	200/85378	843/305980	1800/680835
Unadjusted model	1 (Reference)	1.29 (1.04-1.59)	1.39 (1.25-1.54)	1.24 (1.16-1.33)
Basic model	1 (Reference)	1.29 (1.04-1.59)	1.39 (1.25-1.54)	1.24 (1.16-1.33)
Multivariable model	1 (Reference)	1.14 (0.91-1.43)	1.30 (1.15-1.46)	1.21 (1.11-1.31)
**CVD mortality**				
Cases/person-years	719/1082451	115/85786	410/308146	771/685812
Unadjusted model	1 (Reference)	2.11 (1.53-2.92)	1.86 (1.58-2.19)	1.75 (1.55-1.96)
Basic model	1 (Reference	2.12 (1.53-2.94)	1.83 (1.55-2.16)	1.74 (1.54-1.95)
Multivariable model	1 (Reference)	1.31 (0.91-1.88)	1.21 (0.99-1.47)	1.35 (1.17-1.55)

CVD: cardiovascular disease.

Basic model: adjusted for age and sex.

Multivariable model: adjusted for age, sex, ethnic background, Townsend Deprivation Index, education years, smoking status, alcohol intake, healthy diet score, physical activity, depression, anxiety, eating disorder, hypertension, high cholesterol, diabetes, metformin use, glucocorticoid use and HbA1c.

© 2024 Zhou J et al. *JAMA Network Open.*

eTable 11. Associations of Social Isolation and Loneliness With Risk for All-Cause Mortality in 84 920 People With Different Obesity Classes Compared With 84 920 Matched Control Participants via Multivariable Model

**Outcomes**	**Non-obesity**	**Obesity**		
		**Index≥2**	**Index=1**	**Index=0**
**Social isolation**				
30 kg/m^2≤BMI<35 kg/m^2	1 (Reference)	1.28 (1.09-1.50)	1.23 (1.13-1.34)	0.98 (0.91-1.06)
35 kg/m^2≤BMI<40 kg/m^2	1 (Reference)	1.51 (1.14-2.00)	1.24 (1.06-1.46)	1.19 (1.02-1.39)
BMI≥40 kg/m^2	1 (Reference)	1.90 (1.25-2.89)	2.06 (1.54-2.76)	1.03 (0.78-1.36)
**Loneliness**				
30 kg/m^2≤BMI<35 kg/m^2	1 (Reference)	1.05 (0.86-1.28)	1.15 (1.04-1.27)	1.08 (1.01-1.16)
35 kg/m^2≤BMI<40 kg/m^2	1 (Reference)	1.48 (1.08-2.03)	1.22 (1.01-1.46)	1.23 (1.07-1.41)
BMI≥40 kg/m^2	1 (Reference)	2.05 (1.24-3.38)	1.48 (1.07-2.06)	1.41 (1.10-1.81)

Multivariable model: adjusted for age, sex, ethnic background, Townsend Deprivation Index, education years, smoking status, alcohol intake, healthy diet score, physical activity, depression, anxiety, eating disorder, hypertension, high cholesterol, diabetes, metformin use, glucocorticoid use and HbA1c.

© 2024 Zhou J et al. *JAMA Network Open.*

eTable 12. Association of Individual Components of Social Isolation and Loneliness With Risk of All-Cause Mortality and Cause-Specific Mortality in 84 920 People With Obesity Compared With 84 920 Matched Control Participants via Multivariable Model

**Indicators**	**Non-obesity**	**Obesity**					
		**All-cause mortality**		**Cancer mortality**		**CVD mortality**	
		**Index=1**	**Index=0**	**Index=1**	**Index=0**	**Index=1**	**Index=0**
**Social isolation indicators**							
Live alone	1 (Reference)	1.35 (1.23-1.49)	1.09 (1.03-1.15)	1.33 (1.16-1.52)	1.20 (1.11-1.30)	1.85 (1.46-2.34)	1.18 (1.03-1.34)
Frequency of friend/family visits	1 (Reference)	1.41 (1.22-1.63)	1.12 (1.06-1.18)	1.37 (1.11-1.70)	1.22 (1.13-1.31)	1.86 (1.33-2.62)	1.25 (1.11-1.42)
Leisure/social activities	1 (Reference)	1.26 (1.16-1.36)	1.09 (1.03-1.16)	1.29 (1.15-1.44)	1.20 (1.11-1.30)	1.45 (1.19-1.76)	1.24 (1.08-1.42)
**Loneliness indicators**							
Lonely	1 (Reference)	1.25 (1.13-1.38)	1.11 (1.06-1.18)	1.22 (1.06-1.40)	1.23 (1.14-1.33)	1.40 (1.10-1.79)	1.28 (1.12-1.45)
Able to confide	1 (Reference)	1.13 (1.03-1.24)	1.14 (1.08-1.21)	1.27 (1.11-1.44)	1.22 (1.13-1.31)	1.13 (0.92-1.39)	1.37 (1.19-1.56)

CVD: cardiovascular disease.

Multivariable model: adjusted for age, sex, ethnic background, Townsend Deprivation Index, education years, smoking status, alcohol intake, healthy diet score, physical activity, depression, anxiety, eating disorder, hypertension, high cholesterol, diabetes, metformin use, glucocorticoid use and HbA1c.

© 2024 Zhou J et al. *JAMA Network Open.*

eTable 13. Associations of Social Isolation and Loneliness With Risk of All-Cause Mortality and Cause-Specific Mortality in 93 037 People With Obesity via Multivariable Model After Excluding Participants Who Died During the First 2 Years of Follow-Up

**Outcomes**	**Obesity**			
	**Index≥2**	**Index=1**	**Index=0**	***P*-trend**
**Social isolation**				
All-cause mortality	1 (Reference)	0.86 (0.79-0.92)	0.75 (0.70-0.82)	<0.001
Cancer mortality	1 (Reference)	0.92 (0.82-1.04)	0.88 (0.78-0.99)	0.030
CVD mortality	1 (Reference)	0.74 (0.63-0.86)	0.65 (0.56-0.76)	<0.001
**Loneliness**				
All-cause mortality	1 (Reference)	0.97 (0.88-1.06)	0.86 (0.79-0.94)	<0.001
Cancer mortality	1 (Reference)	1.10 (0.95-1.28)	1.06 (0.92-1.22)	0.960
CVD mortality	1 (Reference)	0.99 (0.82-1.21)	0.85 (0.71-1.03)	0.010

CVD: cardiovascular disease.

Multivariable model: adjusted for age, sex, ethnic background, Townsend Deprivation Index, education years, smoking status, alcohol intake, healthy diet score, physical activity, depression, anxiety, eating disorder, hypertension, high cholesterol, diabetes, metformin use, glucocorticoid use and HbA1c.

© 2024 Zhou J et al. *JAMA Network Open.*

eTable 14. Associations of Social Isolation and Loneliness With Risk of All-Cause Mortality and Cause-Specific Mortality in 84 920 People With Obesity Compared With 84 920 Matched Control Participants Multivariable Model After Excluding Participants Who Died During the First 2 Years of Follow-Up

**Outcomes**	**Non-obesity**	**Obesity**		
		**Index≥2**	**Index=1**	**Index=0**
**Social isolation**				
All-cause mortality	1 (Reference)	1.36 (1.19-1.55)	1.27 (1.18-1.37)	1.02 (0.95-1.09)
Cancer mortality	1 (Reference)	1.29 (1.07-1.57)	1.33 (1.20-1.48)	1.18 (1.07-1.29)
CVD mortality	1 (Reference)	1.76 (1.28-2.41)	1.46 (1.20-1.76)	1.09 (0.93-1.28)
**Loneliness**				
All-cause mortality	1 (Reference)	1.23 (1.04-1.44)	1.18 (1.08-1.29)	1.12 (1.05-1.19)
Cancer mortality	1 (Reference)	1.15 (0.91-1.46)	1.32 (1.17-1.49)	1.22 (1.12-1.33)
CVD mortality	1 (Reference)	1.26 (0.86-1.83)	1.23 (1.00-1.51)	1.30 (1.12-1.51)

CVD: cardiovascular disease.
Multivariable model: adjusted for age, sex, ethnic background, Townsend Deprivation Index, education years, smoking status, alcohol intake, healthy diet score, physical activity, depression, anxiety, eating disorder, hypertension, high cholesterol, diabetes, metformin use, glucocorticoid use and HbA1c.

© 2024 Zhou J et al. *JAMA Network Open.*

eTable 15. Associations of Social Isolation and Loneliness With Risk of All-Cause Mortality and Cause-Specific Mortality in 93 357 People With Obesity via Multivariable Model With All Missing Covariate Data Imputed Using Multiple Imputation

**Outcomes**	**Obesity**			
	**Index≥2**	**Index=1**	**Index=0**	***P*-trend**
**Social isolation**				
All-cause mortality	1 (Reference)	0.84 (0.78-0.90)	0.72 (0.67-0.78)	<0.001
Cancer mortality	1 (Reference)	0.91 (0.81-1.02)	0.86 (0.77-0.96)	0.008
CVD mortality	1 (Reference)	0.73 (0.63-0.84)	0.61 (0.53-0.71)	<0.001
**Loneliness**				
All-cause mortality	1 (Reference)	0.96 (0.88-1.05)	0.85 (0.78-0.93)	<0.001
Cancer mortality	1 (Reference)	1.10 (0.95-1.28)	1.07 (0.93-1.22)	0.888
CVD mortality	1 (Reference)	0.96 (0.79-1.15)	0.83 (0.69-0.99)	0.004

CVD: cardiovascular disease.
Multivariable model: adjusted for age, sex, ethnic background, Townsend Deprivation Index, education years, smoking status, alcohol intake, healthy diet score, physical activity, depression, anxiety, eating disorder, hypertension, high cholesterol, diabetes, metformin use, glucocorticoid use and HbA1c.

© 2024 Zhou J et al. *JAMA Network Open.*

eTable 16. Associations of Social Isolation and Loneliness With Risk of All-Cause Mortality and Cause-Specific Mortality in 84 920 People With Obesity Compared With 84 920 Matched Control Participants via Multivariable Model With All Missing Covariate Data Imputed Using Multiple Imputation

Outcomes	Non-obesity	Obesity		
		Index≥2	Index=1	Index=0
**Social isolation**				
All-cause mortality	1 (Reference)	1.41 (1.24-1.61)	1.30 (1.20-1.39)	1.02 (0.95-1.09)
Cancer mortality	1 (Reference)	1.32 (1.10-1.59)	1.31 (1.18-1.45)	1.15 (1.05-1.26)
CVD mortality	1 (Reference)	1.86 (1.38-2.51)	1.56 (1.30-1.87)	1.11 (0.95-1.30)
**Loneliness**				
All-cause mortality	1 (Reference)	1.25 (1.07-1.46)	1.20 (1.11-1.31)	1.13 (1.06-1.20)
Cancer mortality	1 (Reference)	1.14 (0.91-1.43)	1.30 (1.15-1.46)	1.21 (1.11-1.31)
CVD mortality	1 (Reference)	1.39 (0.97-1.98)	1.24 (1.02-1.51)	1.38 (1.20-1.59)

CVD: cardiovascular disease.

Multivariable model: adjusted for age, sex, ethnic background, Townsend Deprivation Index, education years, smoking status, alcohol intake, healthy diet score, physical activity, depression, anxiety, eating disorder, hypertension, high cholesterol, diabetes, metformin use, glucocorticoid use and HbA1c.

© 2024 Zhou J et al. *JAMA Network Open.*
