# Supplementary material for: Improvement of Social Isolation and Loneliness and Excess Mortality Risk in People With Obesity
Source: JAMA Netw Open. 2024 Jan 22;7(1):e2352824. doi: 10.1001/jamanetworkopen.2023.52824 (PMC10804268; doi:10.1001/jamanetworkopen.2023.52824)
Supplement: Supplement 2. — Data Sharing Statement [file jamanetwopen-e2352824-s002.pdf]

# Data Sharing Statement

Zhou. Improvement of Social Isolation and Loneliness and Excess Mortality Risk in People With Obesity. *JAMA Netw Open*. Published January 22, 2024.
doi:10.1001/jamanetworkopen.2023.52824

**Data**
**Data available:** No
